# Resveratrol Oligomers for the Prevention and Treatment of Cancers

**DOI:** 10.1155/2014/765832

**Published:** 2014-03-23

**Authors:** You-Qiu Xue, Jin-Ming Di, Yun Luo, Ke-Jun Cheng, Xing Wei, Zhi Shi

**Affiliations:** ^1^Department of Cell Biology & Institute of Biomedicine, College of Life Science and Technology, Jinan University, Guangzhou, Guangdong 510630, China; ^2^Department of Urology, Third Affiliated Hospital of Sun Yat-sen University, Guangzhou, Guangdong 510630, China; ^3^Chemical Biology Center, Lishui Institute of Agricultural Sciences, Lishui, Zhejiang 32300, China

## Abstract

Resveratrol (3,4′,5-trihydroxystilbene) is a naturally derived phytoalexin stilbene isolated from grapes and other plants, playing an important role in human health and is well known for its extensive bioactivities, such as antioxidation, anti-inflammatory, anticancer. In addition to resveratrol, scientists also pay attention to resveratrol oligomers, derivatives of resveratrol, which are characterized by the polymerization of two to eight, or even more resveratrol units, and are the largest group of oligomeric stilbenes. Resveratrol oligomers have multiple beneficial properties, of which some are superior in activity, stability, and selectivity compared with resveratrol. The complicated structures and diverse biological activities are of significant interest for drug research and development and may provide promising prospects as cancer preventive and therapeutical agents. This review presents an overview on preventive or anticancer properties of resveratrol oligomers.

## 1. Introduction

There are growing interests in using natural compounds as potential cancer therapeutics or cancer preventive agents for human diseases. Lots of epidemiological data illustrate that there is a significant correlation between dietary intake and incidence of many kinds of cancers, and the incidence of cancer trends to raise year by year in the world due to changes in modern lifestyles and diet custom [1–3]. Due to the unsatisfied effectiveness of current cancer chemotherapy, there is an urgent need of new anticancer drugs with high efficiency and low toxicity. Fighting cancers with novel natural products, especially those extracted from plants-derived diet, seems to be a fascinating strategy. Furthermore,* in vivo* and* in vitro* studies show that many dietary substances have anticancer properties [4, 5]. Resveratrol and its oligomers belong to such kind of dietary substances.

Resveratrol (3,4′,5-trihydroxystilbene) was first isolated from the roots of white hellebore,* Veratrum grandiflorum O. Loes *[6], found in at least 72 plant species including 12 families and 31 genera [7], and widely exist in edible foods and beverages such as mulberries, peanuts, grapes, and red wine [8]. Resveratrol can be classified either as a polyphenol or stilbene and is produced by plants to protect themselves against damage or infection in response to stresses such as heat, insects, bacteria, and fungus [9]. During the last decade, resveratrol attracted increasing attention due to its preventive potential towards the most severe contemporary human diseases, such as cancer, neurodegenerative disease, vascular disease, cardiovascular disease, and aging [10–13]. Resveratrol was reported to make a great influence on the process of carcinogenesis by affecting cancer initiation and progression [8, 14]. Resveratrol was shown to exert a different inhibition to various human tumors cells by* in vitro* experiments through multiple mechanisms as well as different* in vivo* animal models [15, 16]. In addition, there was no significant toxicity to mice after the daily oral administration of high doses of resveratrol for 28 days [17].

**Figure 1:**
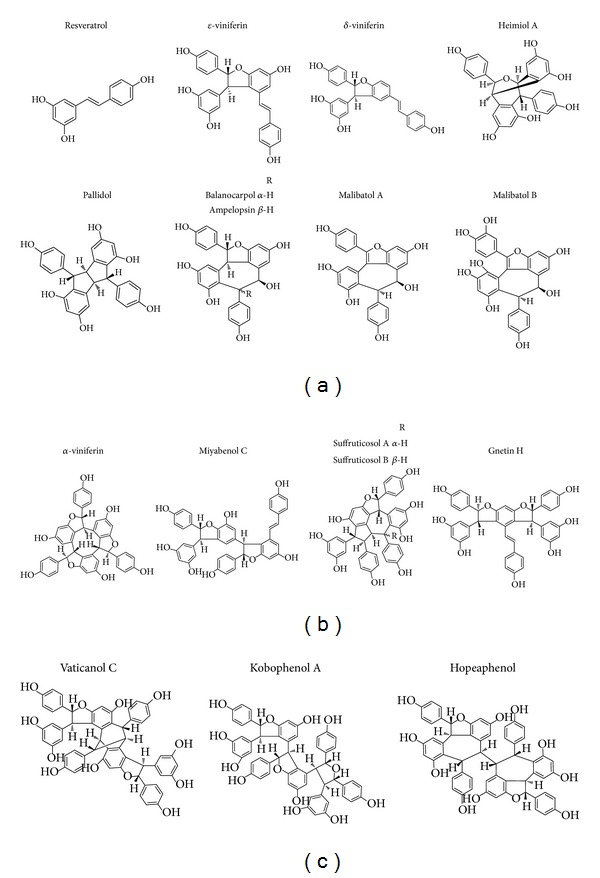
Structure of selected resveratrol oligomers. (a) Resveratrol and resveratrol dimers. (b) Resveratrol trimers. (c) Resveratrol tetramers.

**Figure 2:**
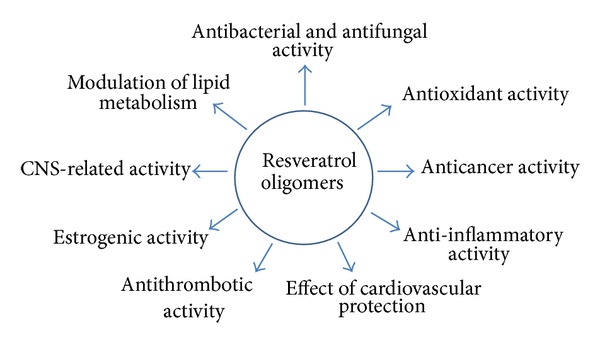
Bioactivities of resveratrol oligomers.

**Figure 3:**
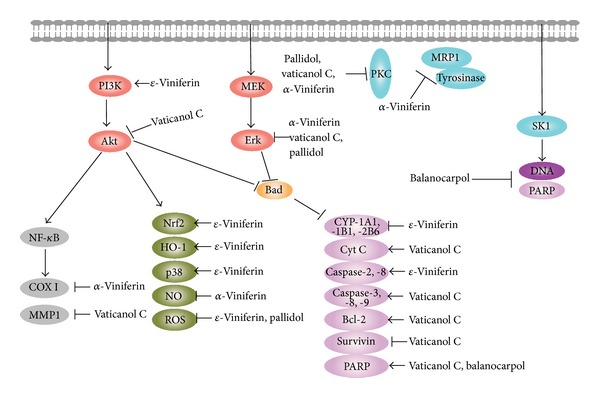
The potential molecular mechanism of resveratrol oligomers for the prevention and treatment of cancer.

Chemical structure analysis showed that resveratrol was a polyphenol biphenyl, and multiple hydroxyl groups affected its biological activities as well as* cis*- or* trans*-structures [18, 19]. In addition to resveratrol, scientists also focus on its derivatives, such as resveratrol oligomers. Resveratrol oligomers are characterized by the polymerization of two to eight resveratrol units and even more and are the largest group of oligomeric stilbenes (Figure 1) [20]. Resveratrol oligomers polyphenols were mainly isolated from five plant families, namely, Vitaceae, Leguminosae, Gnetaceae, Dipterocarpaceae, and Cyperaceae [20–23]. In addition, resveratrol oligomers were recognized as fungal detoxification products of resveratrol metabolism. These oligomers were found to exhibit widely biological activities, such as antibacterial, antifungal, anticancer, anti-HIV, and antioxidant activities (Figure 2) [24, 25]. Their intricate structures and diverse biological activities are of significant interests for drug research and development and may provide promising prospects as cancer preventive and therapeutical agents [26]. Although lots of studies showed various biochemical and pharmacological properties of resveratrol oligomers, so far there is no systematic review about these compounds. In this review, we summarize the recent progresses of the preventive and anticancer activities as well as related mechanisms of resveratrol oligomers (Tables 1 and 2 and Figure 3) and contemplate their prospects as preventive and anticancer agents.

## 2. Resveratrol Dimers

Resveratrol dimers are formed from two resveratrol monomers by oxidation reaction. Series of combination of monomers form different dimers which possess various activities.

### 2.1. *ε*-Viniferin

*ε*-Viniferin, first isolated from* Vitis vinifera *(Vitaceae), is classified as a model for its biosynthesis from resveratrol [21]. Similar to resveratrol, *ε*-viniferin also attracted attention as a phytoalexin and was reported to have antifungal, antibacterial, and antiviral activities [59]. To date, many studies of *ε*-viniferin are about the antioxidant and anticancer activities. The antioxidant activity of *ε*-viniferin is essential in the prevention of oxidative damage or chemical-induced cancer by inhibiting cancer initiation and progression [8, 60]. It was reported that *ε*-viniferin showed the better antioxidant properties to O_2_^−^ radicals (IC_50_ value of 0.12 to 0.16 mM) than resveratrol (IC_50_ value of 0.92 to 0.98 mM) and could inhibit reactive oxygen species production [38, 61]. Cytochromes P450 (CYP) are important oxidative enzymes which metabolize xenobiotics including chemical carcinogens. Therefore, one method of cancer prevention is to inhibit carcinogens activation with inhibitors of these phase I enzymes. Modulation of those enzymes has a great influence on toxicity and carcinogenesis.  *ε*-Viniferin displayed a more potent inhibitory effect than resveratrol for CYP enzymes including CYP1A1, CYP1A2, CYP1B1, CYP2A6, CYP2B6, CYP2E1, CYP3A4, and CYP4A (Ki 0.5 to 20 *μ*M versus 10 to 100 *μ*M, resp.), and this effect was not due to an inhibition of the nicotinamide adenine dinucleotide phosphate reductase [39]  .  *ε*-Viniferin could also regulate the phase II enzymes to induce carcinogen detoxification. There was a report that *ε*-viniferin had an inhibitory effect on noradrenaline and 5-hydroxytryotamine uptake by synaptosomes from rat brain and on the monoamine oxidase activity [40].

*ε*-Viniferin also showed the direct cytotoxicity to various cancer cells [41, 42]. It was reported that *ε*-viniferin could kill C6, Hep G2, HeLa, and MCF-7 cancer cell lines in a dose-dependent manner with IC_50_ values of 18.4, 74.3, 20.4, and 44.8 *μ*g/mL, respectively [43]. In contrast, resveratrol showed stronger cytotoxicity against C6 and Hep G2 with IC_50_ values of 8.2 and 11.8 *μ*g/mL and weaker cytotoxicity against HeLa and MCF-7 with IC_50_ values of 20.4 and 44.8 *μ*g/mL, respectively [44]. In addition, *ε*-viniferin showed a potent anticancer activity against allografted sarcoma S-180 cells in mice and exerted antiproliferative as well as proapoptotic effects on leukemic cells [27, 28, 44]. As for a panel of lymphoid and myeloid cell lines, including U266, RPMI8226, Jurkat, K562 and U937, *ε*-viniferin, and resveratrol both exert the antiproliferative and proapoptotic effect [28]. Further studies on the multiple myeloma cell line U266 showed that *ε*-viniferin and resveratrol could regulate cell cycle by affecting different targets [28]. In this model, *ε*-viniferin induced apoptosis by arresting cell cycle in G2/M, whereas cells treated with resveratrol were accumulated in S phase, and both of them induced apoptosis in a caspase-dependent manner by disrupting normal mitochondrial membrance potential [27, 28]. *ε*-Viniferin was also able to inhibit Hep G2 cell proliferation by blocking cell cycle at G2/M phase [60]. In human colon cancer cell lines, *ε*-viniferin was reported to slightly inhibit cells proliferation; however, resveratrol could inhibit cells proliferation and arrest cell cycle at S phase [62, 63]. Interestingly, the acetylated forms of   *ε*-viniferin and resveratrol possessed more powerful anticancer effects than *ε*-viniferin and resveratrol [62], and this may pave a new avenue to search new cancer-preventive agents from resveratrol derivative or resveratrol oligomers analogs.

*δ*-Viniferin, a isomer of  *ε*-viniferin, only exists in plants with a quite low content. There were only few reports about its chemical synthesis, which limited researches on its biological activities. However, *δ*-viniferin was shown to inhibit the cyclooxygenase-1 and -2 activities with IC_50_ values 5 *μ*M [29, 64].

### 2.2. Pallidol

Pallidol, first isolated from* Cissus pallida*, is a natural ingredient of grape present in red wine at a level equal to that in resveratrol [65]. Pallidol was reported to show stronger antioxidant activity than resveratrol [66] and is a potent and selective singlet oxygen quencher in aqueous system. Reactive oxygen species (ROS), including singlet oxygen ^1^O_2_, superoxide anion  O_2_^∙−^, and hydroxyl radical ^∙^OH, were reported as being important agents causing aging and various human diseases, such as cancer, autoimmune disease, and Parkinson's disease [67]. Pallidol is a selective ^1^O_2_  quencher but does not inhibit O_2_^∙−^ or  ^  ∙^OH. ^1^O_2_ [68] is an excited form of molecular oxygen and usually emerges in photo-sensitized oxidations in biological systems with the ability to react with various targets such as DNA and RNA [69]. Pallidol has a potent ^1^O_2 _quenching effect at low concentration. Therefore, it may be as a pharmacological agent in singlet oxygen-mediated diseases [68]. Additionally, it was reported that pallidol showed the inhibition of cell growth in a time-dependent manner similar to resveratrol in human colon cancer cells, including HCT-116, HT-29, and Caco-2 cell lines [26]. The peracetylated pallidol possessed strong cytotoxicity against KB, Caki-1, 1A9, MCF-7, and HCT-8 cell lines with IC_50_ values ranging from 1.6 to 8.0 *μ*M [45]. Analysis of structure-activity relationship revealed that peracetylated derivatives could increase the anticancer activities of resveratrol oligomers. In addition, pallidol was shown to inhibit protein kinase C activity [30], suppressed the growth of lung cancer cells A549 [46], and exerted effects on 5-hydroxytryptamine 6 receptor-mediated Ca^2+^ responses and extracellular-signal-regulated kinases (ERK)1/2 phosphorylation as 5-hydroxytryptamine 6 receptor antagonists [70].

### 2.3. Balanocarpol

Balanocarpol was isolated from two endemic dipterocarp species* Balanocarpus zeylanicus* (Trimen) and* Hopea jucunda* (Thw.), and the latter is one of the main genuses of* Dipterocarpaceae* to produce varieties of resveratrol oligomers such as balanocarpol, heimiol A, and ampelopsin A, H [47, 48, 71]. Balanocarpol and resveratrol were reported as novel sphingosine kinase 1 (SK1) inhibitors by affecting SK1 expression and cancer cells growth and survival [72]. Balanocarpol was a mixed inhibitor (with sphingosine) of SK1 with  *K*_ic_ = 90 ± 10 *μ*M and *K*_iu_ of ~500 *μ*M, while resveratrol was a competitive inhibitor (with sphingosine) of SK1 with a  *K*_ic_ = 160 ± 40 *μ*M, and both of them could reduce SK1 expression and DNA synthesis and induce poly ADP ribose polymerase (PARP) cleavage in MCF-7 cells [72].

## 3. Resveratrol Trimers

Resveratrol trimers are formed by three resveratrol monomers through head-to-ligation or circular structure, which may lead to their various biological activities. The representative trimers are discussed below.

### 3.1. *α*-Viniferin

*α*-Viniferin is a stilbene trimer isolated from* Caragana sinica*,* Caragana chamlagu,* and the stem bark of* Dryobalanops aromatica* [31]. It was reported that *α*-viniferin could inhibit the activity of some enzymes, such as protein kinase C (PKC) [30, 73], tyrosinase [49], prostaglandin H-2 synthase [50], and acetylcholinesterase [51]. *α*-Viniferin was shown to have inhibitory effect on PKC with IC_50_ values of 62.5 *μ*M* in vitro* [30, 73]. In addition, *α*-viniferin could inhibit 2′,7′-bis-(carboxypropyl)-5(6)-carboxyfluorescein transport mediated by multidrug resistance protein 1 (MRP1) on the human erythrocyte membrane at low concentration [52]. Compared to resveratrol, *α*-viniferin showed 3- to 4-fold higher inhibition on cyclooxygenase activity [50]. *α*-Viniferin also showed significant anti-inflammatory activity on carrageenan-induced paw edema in mice through inhibiting cyclooxygenase-2 effects and nitric oxide synthase [74]. Furthermore, *α*-viniferin powerfully inhibited the signal transducer and activators of transcription 1 (STAT1) inducible inflammatory genes via suppressing ERK-mediated STAT1 activation in interferon-*γ*-stimulated macrophages [75].

*α*-Viniferin displayed a striking growth inhibitory effect on various cancer cell lines. It showed marked cytotoxic activity against HL-60 with IC_50_ values of 2.7 ± 0.5 *μ*M and moderately cytotoxic activity against MCF-7, Hep G2, A549, and murine leukemia P-388 cells [53]. *α*-Viniferin also exerted selective antiproliferative activity against submandibular gland carcinoma but no effects on normal human oral cells such as pulp cells, periodontal ligament fibroblast, and gingival fibroblast [54]. In addition, *α*-viniferin inhibited the proliferation in a concentration- and time-dependent manner by arresting cell cycle at the S phase but not inducing apoptosis of human colon cancer cells* in vitro*, including HCT-116, HT-29, and Caco-2 cell lines, and was more efficient with IC_50_ values ranging from 6 to 40 *μ*M than resveratrol with IC_50_ values ranging from 120 to 170 *μ*M [26]. Together, these studies emphasized the potential of *α*-viniferin for the prevention and treatment of cancer.

### 3.2. Miyabenol C

Miyabenol C, a stilbenoid and natural resveratrol trimer, was reported to possess lots of biological functions. It revealed that miyabenol C could inhibit the activity of rat PKC with IC_50_ values of 27.5 *μ*M, which is similar to *ε*-viniferin [19]. In human lung carcinoma cell lines A549 and NCI-H446, miyabenol C showed cytotoxicity with IC_50_ values of 20 *μ*M and induced apoptosis by inhibiting the effects of PKC isoenzymes [46, 55]. Miyabenol C possessed more potent antiproliferative and proapoptotic effects on different lymphoid and myeloid cell lines with IC_50_ values of 10 to 30 *μ*M than resveratrol with IC_50_ values of 30 to 50 *μ*M, and cells treated with resveratrol and miyabenol C were accumulated in S and G0/G1 phase, respectively [28].

## 4. Resveratrol Tetramers

Resveratrol tetramers are formed from four monomers or two different dimers or a monomer and a trimer, and their complex structures lead to different biological activities.

### 4.1. Vaticanol C

Vaticanol C, isolated from the stem bark of* Vatica rassak* in Dipterocarpaceae, was reported to exert various pharmacological properties, including antiproliferative, antioxidant, anti-inflammatory, and anticancer properties [33, 34, 56, 76]. In a panel of human cancer cell lines, including SW-480, LNCaP, SH-SY5Y, HL-60, and U937, vaticanol C was able to decrease cell viability and showed 4- to 7-fold more potent to induce the death of two cell lines (SW-480 and HL-60) than resveratrol [56]. In another study, the growth of the colon cancer cell lines SW-480, DLD-1, and COLO 201 was significantly inhibited after treated by vaticanol C. The vaticanol C-induced growth inhibition was concentration dependent [33, 56]. Further studies showed that vaticanol C-induced apoptosis was associated with the decrease of mitochondrial membrane potential, release of cytochrome c from mitochondria, and activation of caspases-3 and -9 and could be prevented by overexpression of Bcl-2 [56]. In addition, molecular studies demonstrated that the mechanism of vaticanol C-induced apoptosis was related to the decrease of pErk, pAkt, and pBad [76]. In a mouse model of metastatic mammary carcinoma cells BJMC-3879, the tumor growth was slightly inhibited by vaticanol C, but the multiplicity of metastasis to the lymph nodes and lungs was significantly suppressed due to induced apoptosis with the activation of caspases-3, -8, and -9 by ligand- and death-inducing signaling complex-independent pathway [35, 77]. Recently, there was a report that vaticanol C could activate peroxisome proliferators-activated receptor *α*/*β*/*δ*, and it suggested that vaticanol C could be a novel agent to afford beneficial effects against lifestyle-related diseases [36]. Vaticanol C also showed significant inhibition of matrix metalloproteinase-1 (MMP-1) production [23].

### 4.2. Kobophenol A

Kobophenol A, a natural tetramer of resveratrol, could be isolated from Chinese traditional medicine* Jin Quegen*, the roots of* Caragana sinica Rehd,* and* Caragana chamlagu*. It was reported that kobophenol A possessed the ability to inhibit the activity of PKC [30, 73] and the growth of lung cancer cell line A549 [46] and showed moderate activity against human colon cancer cell lines [26]. Kobophenol A could inhibit acetylcholinesterase activity and display antimicrobial activity on* Staphylococcus aureus *[51]. Additionally, kobophenol A showed the characteristics of selective estrogen receptor modulators and it may be as an agent for the prevention of osteoporosis [78].

### 4.3. Hopeaphenol

Hopeaphenol is a resveratrol tetramer isolated from* Dipterocarpaceae* like* Shorea ovalis* and wines from North Africa [57]. Hopeaphenol inhibited the growth of human cancer cells SW-480 and HL-60 [56] and murine leukemia cells P-388 [32]. It possessed potent cytotoxicity against the human epidermoid carcinoma of the nasopharynx [45], hepatoma [42], and also expounded anti-inflammatory [37], antimicrobial [58], and HIV-inhibitory activities [43].

## 5. Concluding Remarks

Resveratrol widely exists in nature and was extensively studied in clinical trials. However resveratrol oligomers were barely studied due to their rare resource and lacking of studying* in vivo* and in clinical trials. For drug research and design, resveratrol derivatives open a new perspective to selectively develop the health beneficial properties of those natural compounds for the prevention and treatment of human diseases such as cancers. A series of analogs were extracted from different kinds of plants in recent years, which showed more potency for the treatment of human diseases than the parental compound resveratrol. Furthermore, such analogs displayed improved pharmacological properties and various bioactivities, although those results were largely based on experiments with cell cultures or animal studies. There are more and more scientific data to support the use of resveratrol oligomers for human disease prevention or lifespan extension. Resveratrol oligomers target a wide range of molecules that influence cell proliferation, apoptosis, and metastasis. Although the preventive and anticancer mechanism of resveratrol oligomers cannot be limited to a specific pathway, protein, or gene, their use as preventive and anticancer agents has limitless possibilities in its natural and analog forms and should continue to be pursued in future studies.

## Figures and Tables

**Table 1:** The anticancer activities of resveratrol oligomers.

Resveratrol oligomers	Cell lines and inhibition	References
*ε*-Viniferin	C6^+++^, Hep G2^+^, HeLa^++^, MCF-7^+^, HT-29^+^	[27–29]
	U266^++^, RPMI 8226^++^, Jurkat^+^, K562^++^, U937^++^	
Pallidol	A549^+++^	[30]
Balanocarpol	P-388^++^	[31, 73]
*α*-Viniferin	HL-60^+++^, MCF-7^+^, Hep G2^+^, A549^+^, P-388^+^,	[22, 33]
	HCT-116^++^, HT-29^++^, Caco-2^++^	
Miyabenol C	A54^+++^, NCI-H 446^+++^, U266^+++^, RPMI 8226^+++^	[22, 30, 34]
	Jurkat^++^, K562^+++^, U937^+++^	
Vaticanol C	SW-480^+++^, DLD-1^+^, CoLo 201^+^, PC-3^+^, LNCaP^++^	[10, 35–37]
	SH-SY5Y^++^, HL-60^+++^, K562^+^, U937^++^	
Kobophenol A	A549^+^	[30]
Hopeaphenol	SW-480^+^, HL-60^+^, P-388^+++^	[10, 28]

The plus signs indicated the ability to against human cancer cell lines: ^+++^IC_50_ values less of 20 *μ*M; ^++^IC_50_ values range 20 *μ*M to 50 *μ*M; ^+^IC_50_ values of 50 *μ*M to 100 *μ*M.

**Table 2:** The potential targets of resveratrol oligomers involved in apoptosis, cell proliferation, and inflammation.

Resveratrol oligomers	Cell cycle arrest	Induction of apoptosis	Inhibition of proliferation and inflammation	References
*ε*-Viniferin	G2/M	ROS↓, Caspase-2, 8↑, CYP1A1↓, CYP1B1↓, CYP2B6↓	Nrf2↑, HO-1↑, PI3K↑, p38↑	[38–44]
Pallidol	—	ROS↓	PKC↓, ERK↓	[45–48]
Balanocarpol	—	Cleaved PARP↑	DNA↓, SK1↓	[49, 50]
*α*-viniferin	S	—	PKC↓, Tyrosinase↓, MRP1↓, CO*Χ* I↓, NO↓, STAT1↓, ERK↓, IFN-*γ*↓	[45, 51–55]
Miyabenol C	G0/G1	—	PKC↓	[20, 22, 34, 56]
Vaticanol C	—	Caspase-3, 8, 9↑, Bad↑, Cytochrome C↑, Bcl-2↑, Survivin↓, Cleaved PARP↑	MEK↓, Akt↓	[10, 26, 32, 35–37, 57, 58]

The arrows indicate an increase (↑) or decrease (↓) in the levels, activity of the different signals, or phosphorylation status.
